# Synchrotron radiation and X-ray free-electron lasers (X-FELs) explained to all users, active and potential

**DOI:** 10.1107/S1600577521003325

**Published:** 2021-04-27

**Authors:** Yeukuang Hwu, Giorgio Margaritondo

**Affiliations:** a Institute of Physics, Academia Sinica, Taipei 11529, Taiwan; b Department of Engineering Science, National Cheng Kung University, Tainan 70101, Taiwan; c Brain Research Center, National Tsing Hua University, Hsinchu 30013, Taiwan; d Faculté des Sciences de Base, Ecole Polytechnique Fédérale de Lausanne, 1015 Lausanne, Switzerland

**Keywords:** synchrotron, X-FEL, relativity, ponderomotive

## Abstract

Synchrotron radiation evolved over one-half century into a gigantic worldwide enterprise involving tens of thousands of researchers. Initially, almost all users were physicists. But now they belong to a variety of disciplines: chemistry, materials science, the life sciences, medical research, ecology, cultural heritage and others. This poses a challenge: explaining synchrotron sources without requiring a sophisticated background in theoretical physics. Here this challenge is met with an innovative approach that only involves elementary notions, commonly possessed by scientists of all domains.

## Abstract 2

A new teaching strategy presents synchrotron sources and free-electron lasers (FELs) in a form accessible to readers without a theoretical physics background. This specifically includes the microbunching and amplification mechanisms of FELs.

## 1. Background   

Synchrotron radiation sources and free-electron lasers (Margaritondo, 1988 ▸, 2002 ▸; Winick, 1995 ▸; Willmott, 2011 ▸; Mobilio *et al.*, 2015 ▸; Bordovitsyn, 1999 ▸) are, arguably, the most important practical applications of Albert Einstein’s special relativity (Rafelski, 2017 ▸). Indeed, they exploit relativistic properties to produce electromagnetic radiation in spectral ranges where other emitters are unsatisfactory, most notably X-rays.

Explaining such sources to non-physicists is not easy. We propose here an approach that only requires a few basic scientific notions.

### 1.1. Why bother?   

Before starting, we must address a question: why should the readers be interested in synchrotron sources and X-ray free-electron lasers (X-FELs)? This question has two aspects. First, what makes X-rays very important? Second, why should a user of synchrotron or X-FEL sources learn how they work, rather than using them as ‘magic boxes’ emitting the radiation that he/she needs?

The answer to the first question is very general: X-rays are as important as the things in nature that they can probe. These, in turn, are determined by their two ‘sizes’, the physical one (the wavelength) and the energy of their photons.

The X-ray wavelengths are in the same range as the lengths of chemical bonds. And the X-ray photon energies overlap the binding energies of ‘valence’ and ‘core’ electrons in solids and molecules. These are the electrons that are directly involved in the formation of chemical bonds, or indirectly affected by this formation.

In summary, X-rays are ideal probes of chemical bonds. And chemical bonds are the foundation of most research topics in science and technology. This is why X-rays are so very important.

Furthermore, ‘hard’ X-rays penetrate deeply into solid systems, probing their internal properties. This is the foundation of medical radiology. And it is also very useful for materials science, chemistry, biology, medical research, the cultural heritage and several other disciplines.

Let us now discuss the notion of using synchrotron sources and X-FELs as ‘magic boxes’, without understanding how they work. This is – unfortunately – the choice of many users. And it is very wrong, like spending a fortune on buying a fantastic Ferrari, and then using it only in the first gear, ignoring how it works. In fact, many outstanding research careers – including those of several Nobel laureates – profited from a good knowledge of advanced X-ray sources, well beyond the ‘magic box’ level.

Delivering this knowledge is our ambition here. But let us review first the minimum required background to profit from our presentation.

### 1.2. The little you should know before starting to read   

(1) Elementary mechanics: the energy changes are related to the work and to the power of the forces. In particular, a magnetic field applies to a moving electron a ‘Lorentz force’ perpendicular to the velocity, which produces no work and cannot change the kinetic energy.

(2) An electromagnetic wave includes both an electric field and a magnetic field *B*_w_, both in transverse directions with respect to the wave propagation and perpendicular to each other, whose magnitudes are related by *E*_w_ = *cB*_w_ (*c* = speed of light). Its emission requires the acceleration of electric charges, the emitted power being proportional to the square of the acceleration.

(3) An electromagnetic wave with wavelength λ propagating along the direction *z* is described by wavefunctions of the form
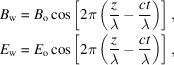
where *B*_o_ and *E*_o_ are the field amplitudes. The wave intensity is proportional to 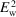 (and therefore to 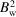 = 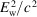).

(4) Einstein’s special relativity is based on two postulates: (i) the speed of light is the same in two reference frames moving with constant velocity with respect to each other; (ii) no experiment can detect a relative motion at constant velocity of two reference frames.

The relativistic properties relevant to synchrotron sources do not need to be known in advance of our presentation, as they will be introduced as required. This specifically refers to the Lorentz transformation, the Lorentz contraction, the Doppler shift and the relativistic beaming, the relativistic mass and its longitudinal counterpart.

(5) Heisenberg’s uncertainty principle for the position and the momentum: the minimum value of the product of the uncertainties is of the order of Planck’s constant.

### 1.3. Initial steps   

**Figure 1:**
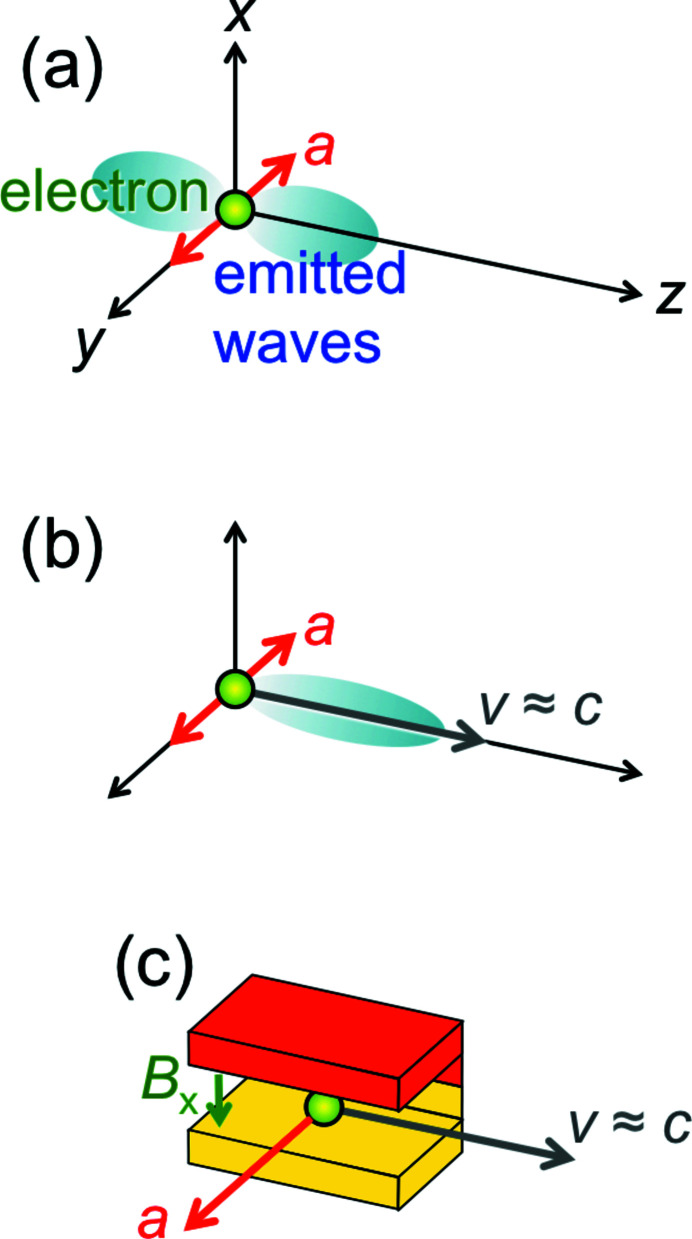
(*a*) Generic mechanism for the emission of electromagnetic radiation, including X-rays: an electron has an acceleration *a* in the transverse direction *y* and emits waves in the longitudinal direction *z*. (*b*) To exploit relativity and produce short X-ray wavelengths, we add a longitudinal velocity *v* close to the speed of light. (*c*) A practical way to implement the mechanism (*b*), using a ‘bending magnet’ (Margaritondo, 1988 ▸) with a magnetic field of magnitude *B*_*x*_ in the transverse *x*-direction, which causes a Lorentz force along *y*.

The emission of electromagnetic radiation in a given (‘longitudinal’) direction [the *z*-axis in Fig. 1 ▸(*a*)] is caused by the acceleration of electrically charged particles in a (‘transverse’) direction perpendicular to it. The acceleration is proportional to the force divided by the mass. Therefore, a synchrotron source uses electrons (or positrons), since their mass is small.

The difficulty of specifically producing X-rays boils down to this. The accelerating device should change the acceleration over distances comparable with the very short X-ray wavelengths – typically on the scale of angstroms, *i.e.* of atoms. Therefore, one should build devices also on the scale of atoms, which is impossible.

Relativity provides a solution by practically ‘shrinking’ the wavelengths (Margaritondo, 1988 ▸, 2002 ▸; Winick, 1995 ▸; Willmott, 2011 ▸; Mobilio *et al.*, 2015 ▸; Bordovitsyn, 1999 ▸). The emitting devices can thus be built on a technologically accessible macroscopic scale. And they exploit relativity by acting on high-energy electrons that move longitudinally with speeds *v* close to the speed of light *c*, as shown in Fig. 1 ▸(*b*).

**Figure 2:**
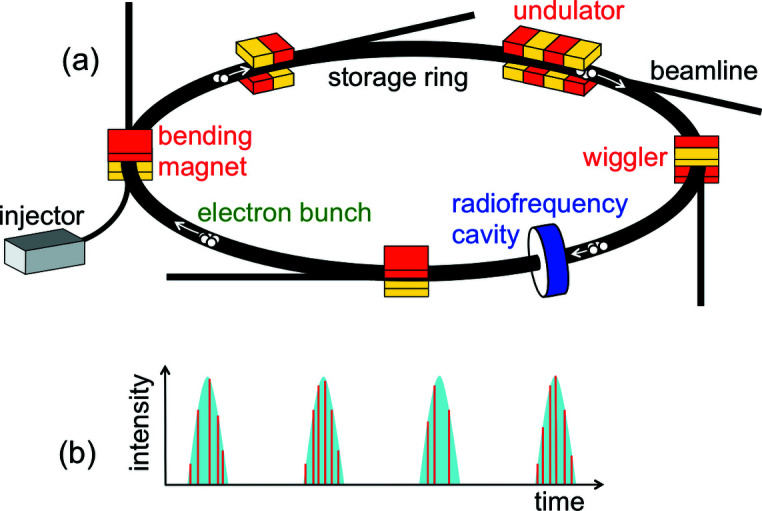
(*a*) Generic scheme of a synchrotron radiation facility with its accelerator (storage ring), the electron injector, a radiofrequency cavity, and X-ray sources of different types with their beamlines. The electrons circulate in the ring as regularly spaced bunches. (*b*) Each time an electron bunch passes through a source, it emits a pulse of radiation, which includes micropulses caused by individual electrons.

A synchrotron facility includes, therefore, a particle accelerator that produces relativistic electrons, plus macroscopic devices that accelerate them in transverse directions – see Fig. 2 ▸(*a*). In the early years of this domain, the accelerators were synchrotrons, hence the name ‘synchrotron radiation’. Now they are ‘storage rings’, but the original name is still universally used.

## 2. Undulators   

**Figure 3:**
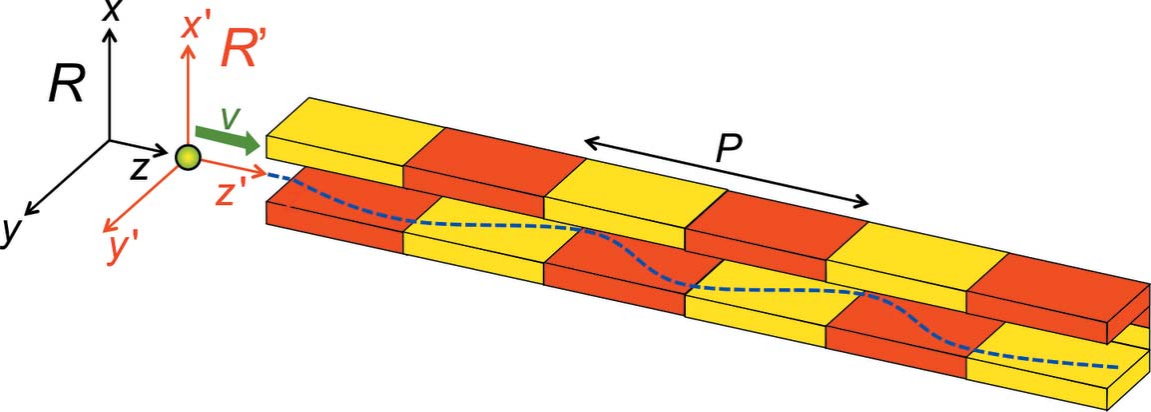
An ‘undulator’, a series of magnets of period *P* in the longitudinal direction, which forces the electrons to oscillate in a transverse direction (Margaritondo, 1988 ▸, 2002 ▸; Winick, 1995 ▸; Willmott, 2011 ▸; Mobilio *et al.*, 2015 ▸; Bordovitsyn, 1999 ▸). Our relativistic description of the consequent undulator emission uses the reference frames R (laboratory and undulator) and R′ (electron).

To understand how synchrotron radiation sources work, we shall analyze a specific example: an ‘undulator’. This is a periodic longitudinal series of magnets (Fig. 3 ▸) that apply Lorentz forces to the relativistic electrons. Such forces cause the electrons to slightly oscillate in a transverse direction. The related acceleration results in the emission of radiation, whose wavelength is related to the undulator period *P*. But this relation is not trivial.

To analyze it, we shall use the two reference frames of Fig. 3 ▸: first, the frame R of the laboratory with the longitudinal coordinate *z* and the transverse coordinates *x* and *y*. Second, the frame R′ of the moving electron with its coordinates *x*′, *y*′, z′. The R′ -frame moves with respect to the R-frame along the *z*-direction, with (longitudinal) speed *v*.

In the R-frame of the laboratory, the periodic magnetic field of the undulator can be written as
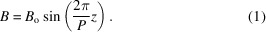
To convert equation (1) to the electron R′ -frame, we must use the relativistic ‘Lorentz transformations’,
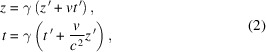
where
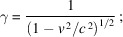
note the second of equations (2): the time is not the same when measured in the two reference frames, contrary to what happens in classical physics. (See Appendix *A* [App appa]: ‘Justifying the Lorentz transformations’.)

We can now use the first of equations (2) to transform the periodic *B*-field of equation (1) to the electron frame R′, obtaining
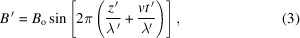
where
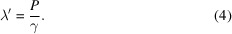
Equation (3) carries two very important messages. First, it looks like a wavefunction traveling with speed *v* in the negative *z*′-direction. Second, the wavelength of this ‘wave’ [equation (4)] is *P*/γ.

These results make sense: the electron, indeed, ‘sees’ the undulator as a traveling periodic magnetic field, a sort of wave. Relativity corroborates this point of view, requiring that in the R′-frame the *B*-field be accompanied by a transverse electric field perpendicular to it, as in an electromagnetic wave.

This electric field is mandated by the second relativistic postulate. Indeed, in the R′-frame the electron has zero velocity, thus the Lorentz force disappears. But this disappearance would reveal the relative motion of the two frames, violating the second postulate. The problem is removed by the appearance of the electric field, whose force replaces the Lorentz force.

The fact that the wavelength equals *P*/γ is also not surprising. This is an example of the so-called ‘Lorentz contraction’: the length of a moving object shrinks by a factor γ along the direction of motion. This effect occurs, in particular, for the length of the undulator and for its period *P*.

Since the electron ‘sees’ the undulator as an electromagnetic wave, it can scatter it back, somewhat like a mirror reflects a beam of light. This backscattering of the undulator ‘wave’ is the basic emission mechanism of synchrotron radiation.

Note that λ′ = *P*/γ is the wavelength in the electron R′-frame. But synchrotron radiation is used in the laboratory, where the electron is a moving source. As a consequence, the wavelength is further decreased by the so-called ‘Doppler shift’.

This phenomenon is commonly detected for sounds. For example, the noises of an approaching train shift to higher frequencies. The frequency is the wave speed divided by the wavelength, so the wavelengths decrease. For electromagnetic waves there is a similar reduction, but relativity makes it very strong: the undulator wavelength of equation (4) is divided by ∼2γ (see Appendix *B* [App appb]: ‘Doppler shift’), becoming
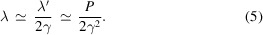



In summary, the combination of two relativistic effects – Lorentz contraction and Doppler shift – decreases the undulator wavelengths by a large factor 2γ^2^, bringing them to the X-ray range.

How large is the factor 2γ^2^? Note that the relativistic mass *m* of the moving electron is related (Rafelski, 2017 ▸) to the rest mass: *m* = γ*m*_0_. Therefore, the most famous of Einstein’s equations, energy = *mc* ^2^, implies that γ = energy/(*m*_0_ *c* ^2^), *i.e.* γ is the energy of the electron measured in terms of its rest energy *m*_0_ *c* ^2^.

The typical energies of electrons in synchrotron sources are several GeV (billions of electronvolts, one electronvolt being the energy given to an electron by a voltage drop of 1 volt). They correspond to γ-values of several thousands. Thus, the contraction of equation (5) is a big effect: for example, γ = 4 × 10^3^ (as ∼2 GeV) shrinks a non-relativistic wavelength of 0.5 cm to ∼1.6 Å.

## 3. Undulators: refinements   

The above analysis can be improved. The γ-factor in equation (5) corresponds to the energy associated with the *longitudinal* relativistic motion of the electron, which determines both the Lorentz contraction and the Doppler shift. The undulator adds a transverse oscillation. But the Lorentz force of the undulator produces no work, so the total kinetic energy remains constant. Thus, the transverse oscillations with velocity *v*_T_ imply a slight decrease of the longitudinal speed and of the related longitudinal γ-factor – and a small increase of the wavelength. In Appendix *C* [App appc] (‘How the electrons move in an undulator’) it is shown that equation (5) must be changed to
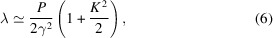
where *K* ^2^ is the square of the so-called ‘undulator parameter’ *K*,
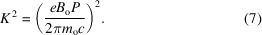
Thus, by changing the magnetic field amplitude *B*_o_ – for example by varying the magnet gap – one can adjust the wavelength as required for specific applications.

**Figure 4:**
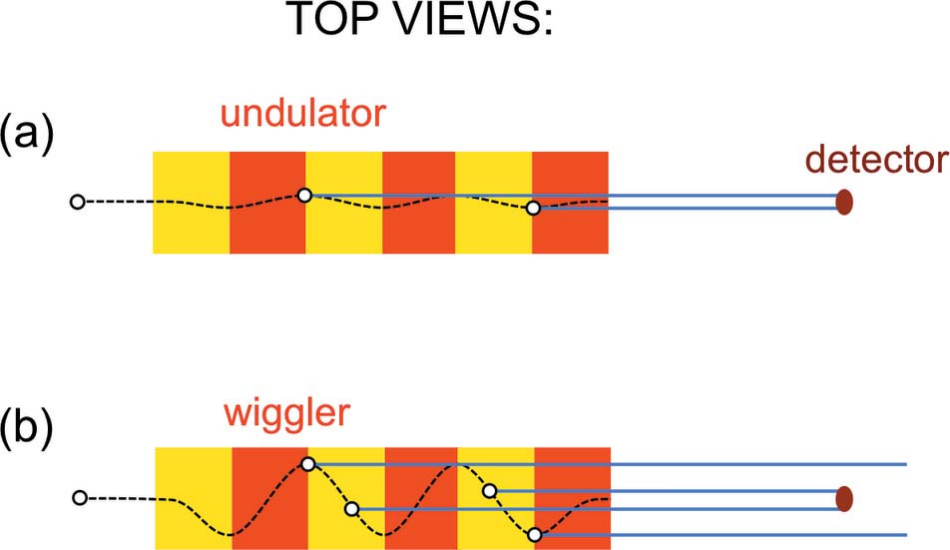
Top views explaining the emission bandwidths of undulators and wigglers. (*a*) During the passage of an electron through an undulator, the collimated beam of synchrotron radiation stays within a small-area detector, since the weak magnetic field causes only small lateral oscillations; this produces a long pulse. (*b*) The larger oscillations in a high-field wiggler bring the emitted beam in and out of the detector, producing a series of short pulses. Note, however, that the transition from undulators to wigglers is not sharply defined. Sometimes, the two terms are used interchangeably: for example, all the insertion devices of free-electron lasers are commonly called ‘undulators’.

Equation (6) is the ‘central’ emitted wavelength. But the undulator also emits a band of wavelengths around this value. The bandwidth is quite narrow, as we can understand from Fig. 4 ▸ – showing the top view of the capture of a narrow undulator beam by a small-area detector.

First, however, why is the emitted beam narrow? This is another aspect of the Doppler effect (Nolte, 2020 ▸)^1^, the relativistic ‘aberration’ (Rafelski, 2017 ▸). Its main consequence is ‘beaming’, *i.e.* the concentration of the radiation to a small angular range.

The phenomenon is similar to what occurs for sound waves emitted by a car, which are ‘projected ahead’ by the source motion when detected from the roadside. But for electromagnetic waves the effect is again boosted by relativity: the emission from a moving source is confined to an exceedingly small angular range ∼2/γ, of the order of milliradians for synchrotron sources. Therefore, the electrons function as extreme ‘flashlights’. (See Appendix *D* [App appd]: ‘Doppler ‘beaming”.)

Let us now go back to Fig. 4 ▸(*a*). The transverse oscillations are weak, so the narrow ‘flashlight’ illuminates the detector during the entire passage of the electron through the undulator. This produces a long pulse. The well known Fourier theorem links the pulse duration to the corresponding wavelength bandwidth: a long pulse implies a narrow band.

What happens if the magnetic field is increased and the electron oscillations are no longer very weak? As shown in Fig. 4 ▸(*b*), they bring the narrow beam in and out of the detector, producing a series of short pulses. The Fourier theorem associates them with a broad bandwidth, around the wavelength of equation (5) [or, better, equation (6)]. Synchrotron sources of this kind are called ‘wigglers’.

Besides undulators and wigglers, there exists a third class of synchrotron sources: the ‘bending magnets’ [Fig. 1 ▸(*c*)]. These are dipole magnets that keep the electrons in closed orbits around the storage ring, and also cause the emission of radiation by accelerating them. The wavelength corresponds to the ‘cyclo­tron frequency’ ω of the circular motion in a constant magnetic field, λ = 2π*c*/ω. For a non-relativistic electron, the Lorentz force has magnitude |*evB*_*x*_| and causes a centripetal acceleration ω*v* = |*evB*_*x*_|/*m*_0_. Thus, ω = |*eB*_*x*_|/*m*_0_, and
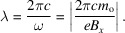
The numerical values from this equation are not X-ray wavelengths, but much longer, typically microwaves. For example, *B*_*x*_ = 1 T gives λ ≃ 1 cm.

But this changes if the electron is relativistic. As mentioned, relativity introduces in the electron R′-frame an electric field that causes a force of magnitude |*ev*γ*B*_*x*_|. This replaces the force |*evB*_*x*_| in the derivation of the wavelength, giving
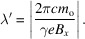
With the 2γ Doppler shift, in the laboratory R-frame the wavelength becomes
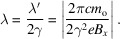
Note the same factor 2γ^2^ as in equations (5) and (6), shifting λ to the X-ray range.

With an analysis similar to Fig. 4 ▸, we can realize that the electron-flashlight passing through a bending magnet produces a short pulse – which the Fourier theorem associates with a broad bandwidth (Margaritondo, 2018 ▸). From it, specific wavelengths can be extracted with a monochromator.

## 4. Brightness, polarization, coherence, time structure   

The previous discussion demonstrates that relativistic electrons can emit short-wavelength radiation. But does this guarantee high-quality X-rays sources? Before responding, we must define what is a ‘good’ source. As for other emitters of radiation, the quality must be assessed using appropriate parameters. The most important (Margaritondo, 1988 ▸, 2002 ▸; Winick, 1995 ▸; Willmott, 2011 ▸; Mobilio *et al.*, 2015 ▸; Bordovitsyn, 1999 ▸) is the ‘brightness’ or ‘brilliance’ *b*.

### 4.1. Brightness   

**Figure 5:**
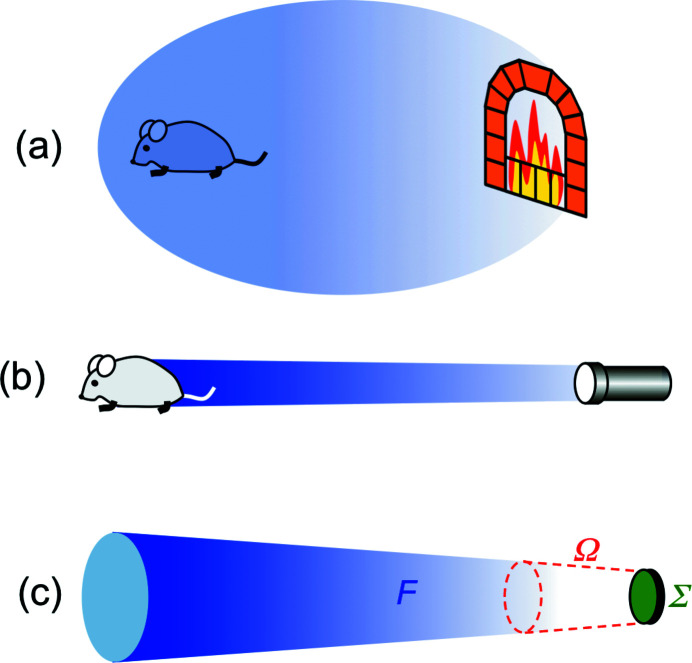
The notion of ‘brightness’ or ‘brilliance’. (*a*) A fireplace does not have high brightness since its emission, coming from a large area and being spread over a broad angular range, cannot bring much radiation into the zone of utilization. (*b*) A flashlight is more effective, *i.e.* it has high brightness. (*c*) We define the brightness (Margaritondo, 1988 ▸, 2002 ▸; Winick, 1995 ▸; Willmott, 2011 ▸; Mobilio *et al.*, 2015 ▸; Bordovitsyn, 1999 ▸) by combining [equation (8)] the emitted flux *F* with the geometric parameters Ω (solid angle of emission) and Σ (source area).

This parameter describes the capacity to bring a high radiation power into the area of utilization. Let us compare (Fig. 5 ▸) a fireplace with a flashlight (or a laser pointer). The fireplace may emit a large flux *F*, but does not have high ‘brightness’: its radiation is not concentrated because it comes from a large emitting area and is spread over a broad angular range. A flashlight, even with a limited flux, reaches high brightness because it emits from a small area and within a small angular range.

Such arguments lead (Margaritondo, 1988 ▸, 2002 ▸; Winick, 1995 ▸; Willmott, 2011 ▸; Mobilio *et al.*, 2015 ▸; Bordovitsyn, 1999 ▸) to the following definition of brightness [Fig. 5 ▸(*c*)],
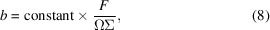
where Ω is the solid angle where the emission occurs and Σ is the source area. Synchrotron radiation boosted (Margaritondo, 1988 ▸) the brightness of X-ray sources since the 1970s by more than 22 orders of magnitude. This is a truly spectacular performance increase – beating by 15 orders of magnitude the much acclaimed power growth of computers!

What allowed such an exceptional improvement? Essentially, four factors, two of which are direct consequences of relativity.

Not linked to relativity is the first one: the use as emitters of ‘free’ electrons in an accelerator. In conventional (not synchrotron) X-ray sources, the electrons are in a solid that can be damaged by excessive emitted power. This is not a problem for the ‘free’ electrons in the vacuum chamber of a high-energy accelerator, which can handle much higher power levels.

The second factor, also not linked to relativity, is the small source size Σ in equation (8). However, we should not naïvely identify Σ with the cross section of an electron. In fact, many electrons circulate in a storage ring along trajectories that are slightly different from each other. The source size Σ corresponds to the transverse cross section of the collective electron beam. The very advanced technology of particle accelerators makes it exceedingly small, boosting *b*.

The third factor is the high flux *F*, which is a straightforward result of relativity. In classical electromagnetism, the power emitted by an accelerated charge (Larmor law) is proportional to the square of the transverse acceleration. The acceleration is a coordinate divided by the square of the time. As we change from R′ to R, the longitudinal relativistic motion of the source does not affect the transverse coordinates. But – see equation (2) – it does multiply the time by 1/γ, the acceleration by γ^2^ and its square by γ^4^.

Therefore, the emitted power and the flux in the laboratory R-frame are proportional to the fourth power of the electron energy. A storage ring brings the electrons to very high energies, and their fourth power yields extremely high fluxes.

Note, however, that γ^4^ = [energy/(*m*_0_ *c* ^2^)]^4^: the emission is inversely proportional to 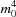. Thus, a small-mass particle like the electron emits much more radiation than a heavy hadron such as a proton, which is difficult to accelerate.

Finally, relativity also enhances *b* by decreasing the solid angular spread Ω with the ‘beaming’. Note, however, that for a bending magnet [Fig. 1 ▸(*c*)] the spread is small only in the vertical direction, whereas in the horizontal direction the emitted beam sweeps over a large angular range.

### 4.2. Polarization   

This is another important and useful property of synchrotron sources. And it is the simplest one to explain.

An electromagnetic wave is a propagating transverse perturbation of the electric and magnetic fields. If the perturbation of each field is limited to only one transverse direction, then the wave is linearly polarized. If the perturbation directions rotate, the wave has circular or elliptical polarization.

**Figure 6:**
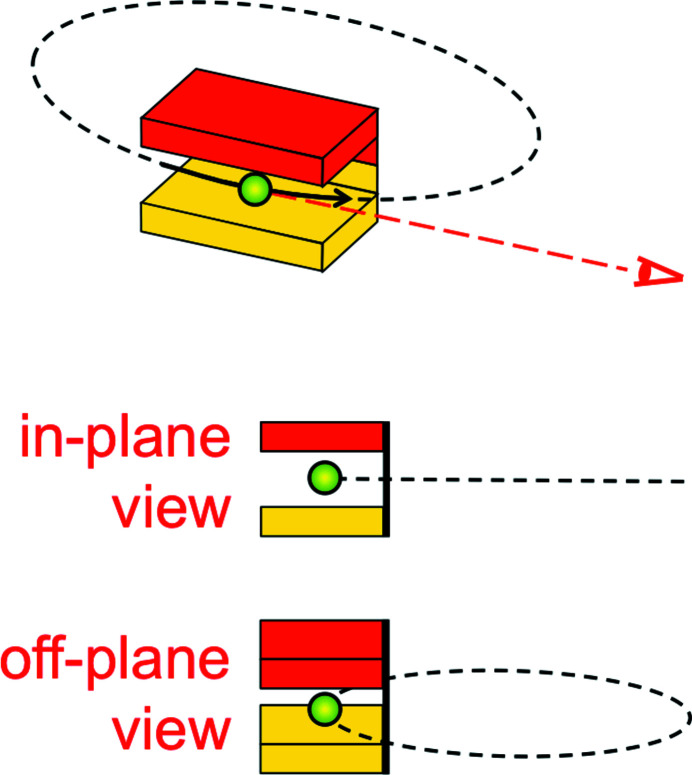
Polarization of synchrotron radiation. Top: a bending magnet causes the electrons to travel along a trajectory (solid line), which is a portion of a circle (dashed line). Middle: seen from the horizontal plane, the circle looks like a straight line, corresponding to linear polarization. Bottom: from a point of view slightly off the horizontal plane, the circle looks like an ellipse, and corresponds to elliptical polarization.

For synchrotron radiation, the perturbation is caused by the magnetic device that induces the transverse electron acceleration. Consider (Fig. 6 ▸, top) a bending magnet that deflects an electron in the horizontal plane, forcing it to move along a portion (solid line) of a circle (dashed line). Seen from the horizontal plane, the circle looks like a straight line. And the corresponding acceleration and electric field perturbation are horizontal.

Therefore, the waves emitted by a bending magnet, when detected in the horizontal plane, are linearly polarized (Fig. 6 ▸, middle). Likewise, one can realize that a planar undulator as that of Fig. 3 ▸ also produces linearly polarized waves.

An observer at an angle off the horizontal plane sees instead the circular trajectory in a bending magnet as an ellipse (Fig. 6 ▸, bottom) – and the wave as elliptically polarized. This is not, however, an efficient way to obtain elliptical polarization. In fact, the relativistic beaming confines the emission to a narrow angular range, so its intensity sharply decreases when detected in directions outside the horizontal plane. To produce intense elliptically polarized synchrotron radiation one must use instead special ‘elliptical undulators’.

### 4.3. Coherence   

Coherence has been for centuries a widely used property in visible-light optics. Its impact on X-ray science is more recent and more limited, but its present expansion (Hwu *et al.*, 1999 ▸; Margaritondo *et al.*, 2008 ▸; Stampanoni *et al.*, 2014 ▸; Munro, 2017 ▸; Chin *et al.*, 2020 ▸) justifies our interest. First, however, we must discover what it is.

In classical physics, X-rays (like visible light) can produce wave-like effects such as interference or diffraction. But these phenomena are rarely observed in everyday life. Why? Because to produce them the radiation must possess, indeed, coherence.

**Figure 7:**
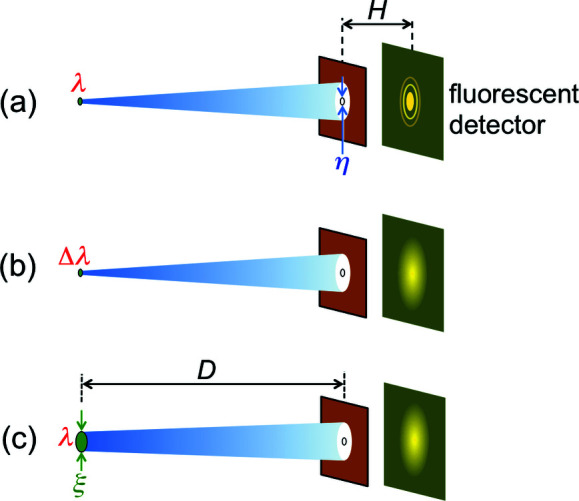
The notion of coherence, introduced using pinhole diffraction. (*a*) A point-like source emitting only one wavelength always produces a visible fringes pattern. (*b*) If the emission is not a single wavelength but a band, the fringes may be washed out. (*c*) Likewise, if the source is not a point but has a finite area, the fringes may not be visible.

To introduce this property, we can use any kind of wave-like phenomenon – for example, the diffraction by a circular pinhole of diameter η shown in Fig. 7 ▸. Fig. 7 ▸(*a*) illustrates the extreme case of a point-like source that emits only one wavelength λ. This source has full coherence: passing through the pinhole its radiation always produces a visible diffraction pattern with a bright central zone surrounded by fainter circular fringes.

The notion of coherence emerges if, instead of a single wavelength λ, the source emits a wavelength band of width Δλ centered at λ [Fig. 7 ▸(*b*)]. Each wavelength in Δλ produces a diffraction pattern, but the superposition of all patterns may wash out the fringes, making diffraction impossible to detect. This leads us to the notion of ‘time coherence’ or ‘longitudinal coherence’.

Time coherence is not the only property required to see wave-like phenomena. In Fig. 7 ▸(*a*), we assumed that the source is point-like, *i.e.* infinitely small. But, in general, a source has a finite size, for example it can be a disk of diameter ξ [Fig. 7 ▸(*c*)]. Each one of its emitting points produces a pattern. And, again, the pattern superposition may wash out the fringes. If it does not, the source has ‘lateral coherence’ or ‘spatial coherence’.

We can discover the conditions for longitudinal and lateral coherence by using the quantum nature of electromagnetic radiation. Indeed, coherence is a quintessential quantum property (Stöhr, 2019 ▸).

The early quantum physics assumed the coexistence of the particle and wave natures for photons (as well as for electrons). But quantum electrodynamics abandoned this notion, considering the electromagnetic radiation as only made of photons. Can photons produce wave-like phenomena, *i.e.* possess coherence? The answer is positive if their size in the relevant direction(s) is larger than the wavelength, so their electromagnetic field (or, better, their probability field) can probe at least one wavelength.

The photon size corresponds to Heisenberg’s position uncertainty, which, in the (longitudinal) direction of the photon propagation, is
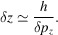
The momentum uncertainty δ*p*_*z*_ can be estimated from the momentum magnitude, *h*/λ,
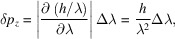
so that δ*z* ≃ λ^2^/Δλ. To observe wave-like phenomena, we must have λ^2^/Δλ > λ, or
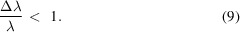
This is the condition for ‘time coherence’ or ‘longitudinal coherence’. And it is not a very stringent one: this is why we can witness wave-like phenomena like the soap bubble fringes. Indeed, our eyes filter the solar light so that the condition of equation (9) is fulfilled.

**Figure 8:**
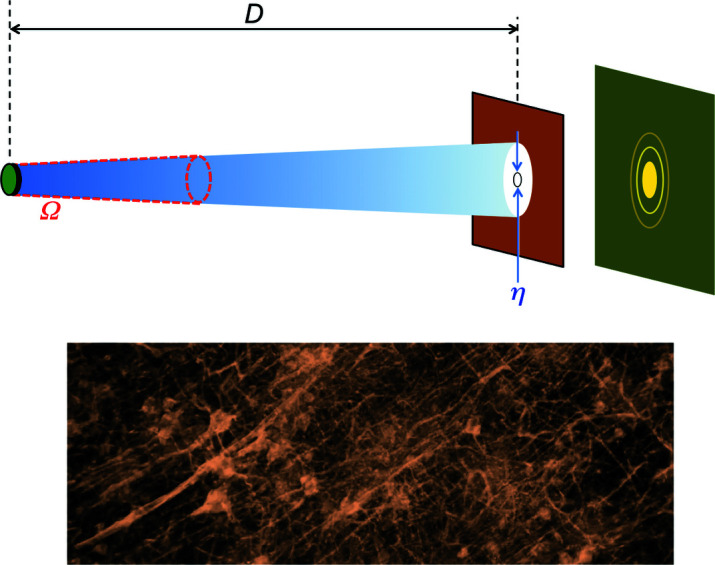
Top: the parameters used to define the ‘coherent power factor’ of equation (11). Bottom: an example of advanced radiology based on coherence – microscopic radiograph of a portion of a neuron network.

Similar simple arguments illustrate lateral coherence in terms of the photon sizes in the transverse directions. Consider Fig. 7 ▸(*c*): if the pinhole diameter is much smaller than ξ, then the uncertainty in the photon directions corresponds to an angle ∼ξ/*D*. As a consequence, the uncertainly in the photon momentum in a transverse direction like *x* is
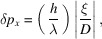
so the Heisenberg’s photon size along *x* is
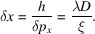
To produce diffraction, this size must be bigger than λ, therefore
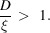
This is a first condition for lateral coherence. Furthermore, the photon size δ*x* cannot be smaller than the diameter η of the pinhole, otherwise photons pass through it as individual particles without producing wave-like phenomena,
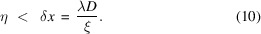
We can elaborate on this result assuming (Fig. 8 ▸, top) that the source emits radiation within a solid angle Ω. Only the portion passing through the pinhole participates to diffraction. The pinhole corresponds to a solid angle (πη^2^/4)/*D* ^2^, so this portion equals
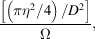
and, according to equation (10), cannot exceed
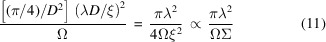
(where the source area Σ equals πξ^2^/4).

The quantity λ^2^/(ΩΣ) in equation (11) is called the ‘coherent power factor’. A source is laterally coherent if it has a large value of this parameter. And this can be obtained not only with a small size but also with a narrow angular spread.

Are synchrotron sources coherent, longitudinally and/or laterally? Longitudinal coherence requires [equation (9)] a narrow bandwidth Δλ. But the bandwidths of bending magnets and wigglers are broad: longitudinal coherence necessitates spectral filtering by a monochromator. This causes a large intensity loss, but the high initial flux makes it manageable. Undulators emit instead narrow bandwidths, guaranteeing a reasonable level of time coherence.

Let us now consider lateral coherence. For many decades after Roentgen’s discovery of X-rays, the sources had a large size and a broad angular range, thus their coherent power [equation (11)] was low. Note that the coherent power is proportional to the square of the wavelength, much smaller for X-rays than for visible light. Therefore, lateral coherence is more difficult to obtain for X-rays. Not surprisingly, many X-ray scientists ignored the interference and diffraction phenomena that were widely exploited for visible light and other types of long-wavelength radiation.

Things changed dramatically with the advent of synchrotron radiation. One should note that the same geometric parameters Σ and Ω define the brightness [equation (8)] and the coherent power factor [equation (11)]. So, the efforts to improve the brightness by decreasing Σ and Ω yielded high lateral coherence as a byproduct. This opened the way to new and powerful X-ray techniques like ‘phase contrast radiography’ (Margaritondo *et al.*, 2004 ▸; Weon *et al.*, 2006 ▸; Stampanoni *et al.*, 2014 ▸; Munro, 2017 ▸) – whose results are very impressive as shown for example by Fig. 8 ▸ (bottom).

Phase contrast radiography is an excellent example of the advantages of knowing how a synchrotron source works rather than using it as a ‘magic box’. This knowledge led to the realization (Margaritondo *et al.*, 2008 ▸; Hwu *et al.*, 1999 ▸) that the technique requires only limited levels of longitudinal and lateral coherence – and is feasible with all generations of synchrotron sources. The consequence was an explosion of the applications of phase contrast radiology.

### 4.4. Time structure   

This is a particularly useful property for time-dependent applications like the analysis of the chemical reaction dynamics. Consider [Fig. 2 ▸(*a*)] the electrons circulating along closed orbits in a storage ring. Around the ring there are several synchrotron sources – bending magnets, wigglers and undulators – delivering radiation to beamlines connected to experimental systems. Passing through a source, an electron can emit a radiation pulse: this causes the basic time structure of synchrotron radiation, but not the most important one.

More interesting is, indeed, the time structure caused by the ‘bunching’ of electrons around the ring. The cause of bunching is the following.

By emitting synchrotron radiation, an electron loses energy. If such energy is not restored, the electron quickly stops circulating around the storage ring. Energy restoration is provided by one or more ‘radiofrequency cavities’, which accelerate the passing electrons with an electric field.

Why are they called ‘radiofrequency cavities’? The typical electron path around the ring is hundreds of meters, and the electron speed is ∼*c* ≃ 3 × 10^8^ m s^−1^. Thus, the circulation time is of the order of 100/(3 × 10^8^) ≃ 0.3 µs: each electron passes through the cavity with a frequency of the order of megahertz. This is, indeed, a radiofrequency.

The electric field must ‘kick’ the electrons precisely when they pass through the cavity. Thus, the only steadily circulating electrons are in bunches synchronized with this field. This influences the time structure: when an electron bunch passes through a synchrotron source (*e.g.* a bending magnet), it emits a radiation pulse. And each pulse contains many micropulses due to the emissions of individual electrons – see Fig. 2 ▸(*b*).

This bunch-related time structure is very useful for a variety of specialized experiments. For example, a synchrotron pulse can trigger a phenomenon at a well defined time, making it possible to study its subsequent evolution.

## 5. From synchrotrons to X-FELs   

The properties of a synchrotron radiation source – such as high brightness, angular collimation and spatial coherence – are quite reminiscent of those of a laser. However, one should not confuse the two kinds of sources: synchrotron emission is not a laser mechanism.

In recent years, advanced X-FELs were realized, whose emission process is somewhat related to lasing (Bonifacio *et al.*, 1994 ▸; Dattoli *et al.*, 1995 ▸; Ribic & Margaritondo, 2012*a* ▸; Ishikawa *et al.*, 2012 ▸). They are the short-wavelength counterparts of infrared FELs, whose realization – pioneered by John Madey – dates back to the early 1970s (Madey, 1971 ▸).

The main similarity between an X-FEL and a standard laser is the use of ‘optical amplification’, *i.e.* the intensity increase of the radiation along the device. In a standard laser, the amplification is caused by stimulated emission and population inversion. In an X-FEL, it is due to the intriguing mechanism called ‘microbunching’ (Bonifacio *et al.*, 1994 ▸; Brau, 1990 ▸; Dattoli *et al.*, 1995 ▸; Margaritondo & Ribic, 2011 ▸; Ribic & Margaritondo, 2012*a* ▸) which we shall discover soon.

One important difference between an X-ray laser and a visible/infrared laser is the use in the latter of an ‘optical cavity’ formed by two mirrors, which lengthens the radiation path to enhance the amplification. Such a cavity does not exist for X-rays due to their low reflectivity. Therefore, the optical amplification in an X-FEL must be strong enough to produce lasing in a single pass: this is called the ‘high gain’ regime.

### 5.1. Microbunching   

**Figure 9:**
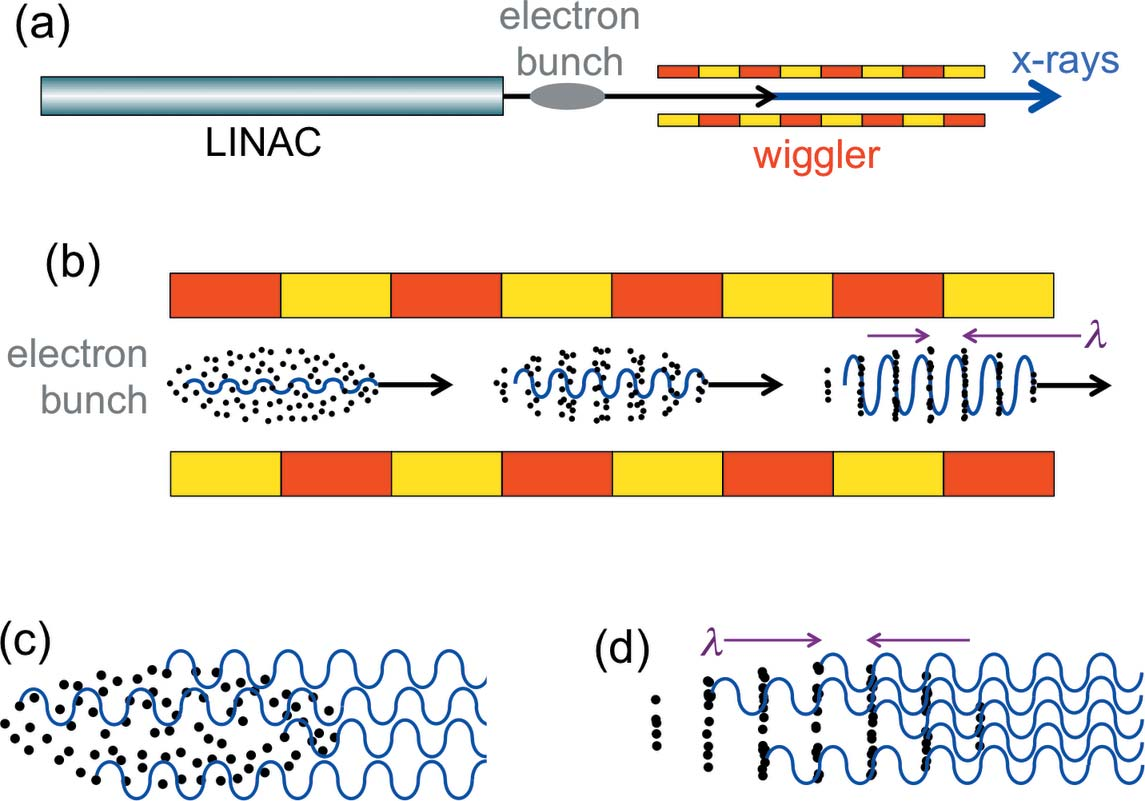
The main components of an X-FEL: (*a*) linear accelerator (LINAC) that produces relativistic electrons, and a long wiggler. (*b*) As a bunch of electrons travels along the wiggler, the interaction with the previously emitted waves progressively creates periodic electron ‘slices’ (‘microbunches’) with a period equal to the wavelength. The waves emitted by microbunched electrons (*d*), unlike those without microbunching (*c*), are correlated and cause optical amplification.

The basic components of an X-FEL [Fig. 9 ▸(*a*)] are a very long wiggler (or undulator) and a LINAC (linear accelerator). The LINAC produces relativistic electrons in bunches. The wiggler has two roles: it forces the emission of radiation by the electrons, and produces the ‘microbunching’ that confines the electrons to thin periodic ‘slices’ with period equal to the wavelength λ.

Consider [Fig. 9 ▸(*b*)] a bunch of electrons entering the X-FEL wiggler: some electrons start, at random, to emit waves. Afterwards, such waves and the electron bunch travel together, interacting with each other. This interaction, which we previously neglected, is the cause of microbunching.

Compare now the wiggler-induced emission of X-rays by electrons without and with microbunching [Figs. 9 ▸(*c*) and 9 ▸(*d*)]. The microbunched electrons emit waves in phase with each other, causing optical amplification.

Full theories of the X-FEL microbunching and optical amplification are very complicated (Bonifacio *et al.*, 1994 ▸; Brau, 1990 ▸; Dattoli *et al.*, 1995 ▸; Margaritondo & Ribic, 2011 ▸; Ribic & Margaritondo, 2012*a* ▸), handling several interacting factors with complex mathematics. But we can grasp some basic facts with simplified arguments.

**Figure 10:**
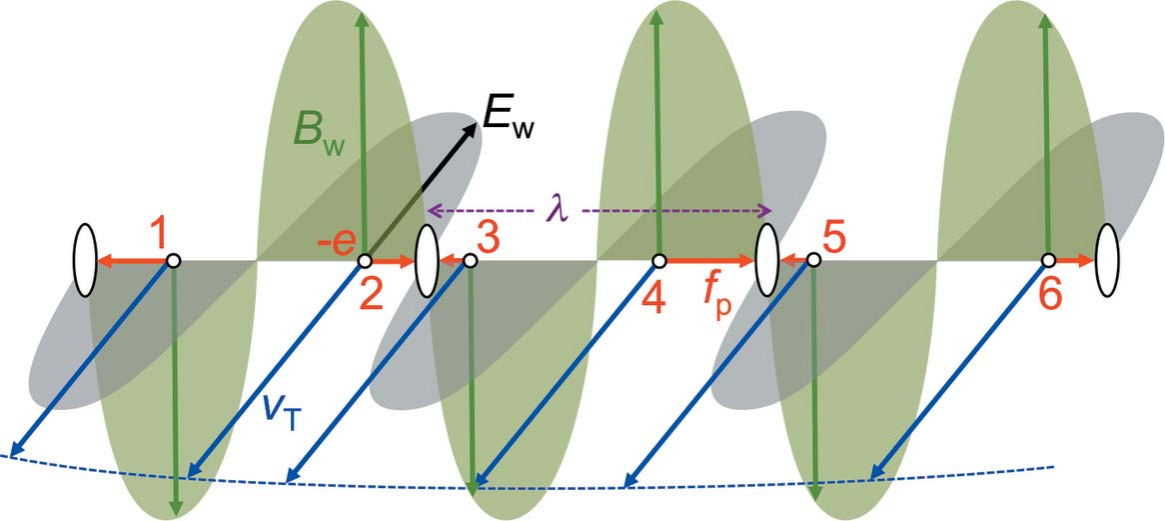
The ‘ponderomotive’ forces *f*_p_, whose magnitudes are formally equivalent [equation (12)] to those of the Lorentz forces caused by the wave magnetic field *B*_w_ and by *v*_T_, the transverse velocity of the wiggler-induced electron undulations. Such forces push some electrons (for example 2, 4, 6) in the forward longitudinal direction and others (1, 3, 5) backwards. But in both cases they accumulate in periodic microbunches, with periodicity equal to the wavelength.

Consider (Fig. 10 ▸) a wave emitted after the electron bunch enters the wiggler, with its (transverse) electric and magnetic fields *E*_w_ and *B*_w_. The interaction between *E*_w_ and the wiggler-induced oscillating transverse velocity produces (Bonifacio *et al.*, 1994 ▸; Brau, 1990 ▸; Dattoli *et al.*, 1995 ▸) the so-called ‘ponderomotive’ forces *f*_p_, a well known notion in plasma physics. These are the forces that shift the electrons towards the microbunches along the longitudinal direction.

This longitudinal effect could be a bit surprising, since both *E*_w_ and the Lorentz forces caused by *v* and *B*_w_ act in transverse directions. But let us have a closer look at the interplay of transverse and longitudinal phenomena.

We already discussed in Section 3 the Lorentz forces caused by an undulator or wiggler *B*-field, and their effects on *v*_T_ and *v*_L_. Let us consider now the transverse force caused by the electric field *E*_w_ of the previously emitted wave. This force slightly changes *v*_T_ and through it the longitudinal Lorentz force caused by *v*_T_ and *B*.

This change corresponds to the ‘ponderomotive’ force. Its magnitude (see Appendix *E* [App appe]: ‘Ponderomotive forces’) is
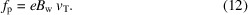
Therefore, the longitudinal ‘ponderomotive’ force is formally equivalent to a Lorentz force caused by *B*_w_ and *v*_T_ (Ribic & Margaritondo, 2012*a* ▸,*b* ▸). Its effects can thus be analyzed in terms of this force.

As seen in Fig. 10 ▸, the ‘ponderomotive’ forces push the electrons longitudinally, either in the forward or backward direction – depending on the relative directions of the vectors *E*_w_ and *v*_T_. But we realize that in both cases the electrons are shifted to microbunches at a distance λ from each other.

There is, however, a subtle point in this mechanism. Imagine that after the situation of Fig. 10 ▸ the electrons and the wave travel together at the same speed, so the wavefields *E*_w_ and *B*_w_ applied to each electron are constant. However, after one-half wiggler period the transverse velocity *v*_T_ is reversed, together with the conditions of Fig. 10 ▸ – and the electrons should be pushed out of their ‘microbunches’, which would be destroyed.

Is this what happens? Actually no: the electrons and the wave *do not* travel together but with slightly different speeds: *v* < *c*. Taking into account this difference (Margaritondo & Ribic, 2011 ▸; Ribic & Margaritondo, 2012*a* ▸,*b* ▸), the conditions of Fig. 10 ▸ are not reversed but stay the same, continuing the microbunching.

In fact, over a distance equal to one-half wiggler period, *P*/2, and therefore to a time *P*/(2*v*) ≃ *P*/(2*c*), an electron ‘slips back’ with respect to the wave by
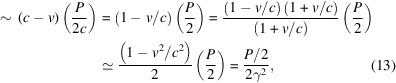
which according to equation (5) is λ/2. This half-wavelength slip means that the wavefields are reversed like *v*_T_, and the relative directions do not change, preserving the conditions for microbunching.

### 5.2. Optical amplification   

**Figure 11:**
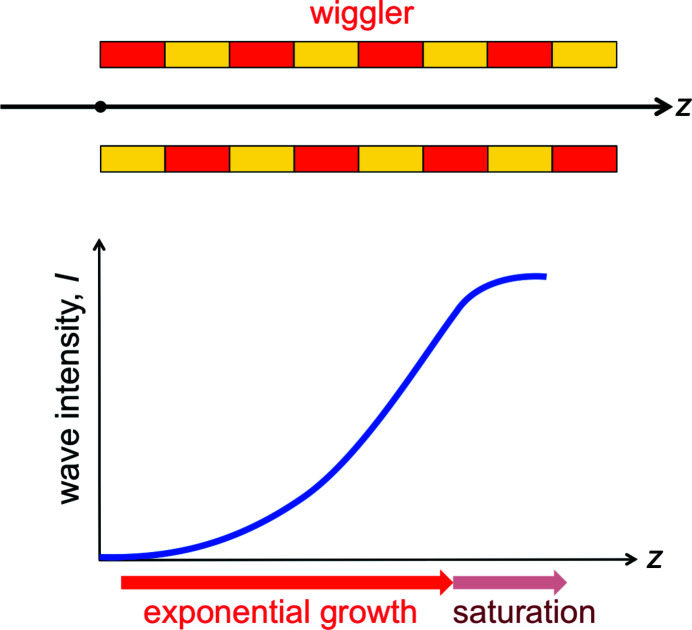
As the electrons enter and then travel along the wiggler (top), after a short initial phase the optical amplification causes an exponential increase of the wave intensity (bottom). But then the increase saturates, as explained in the text.

Let us now see how microbunching influences the wave intensity. After the initial microbunching induced by the first emitted waves, the microbunched electrons continue to be ‘shaken’ by the wiggler and emit radiation in a coordinated way as seen in Fig. 9 ▸(*d*). The experiments show that this leads to the exponential increase of the wave intensity *I* with the distance *z* along the wiggler, as seen in Fig. 11 ▸,
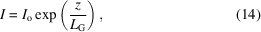
where *L*_G_ is the so-called ‘gain length’. But eventually this growth saturates. Why the exponential increase and why the saturation?

After the initial microbunching, the coordinated emission of microbunched electrons starts to increase the wave intensity. In turn, the enhanced wave strengthens the microbunching and therefore the emission by microbunched electrons, and so on. This causes the exponential increase of equation (14).

Assume, indeed, that the rate of increase of the wave intensity is jointly determined by two factors: (1) the rate of energy transfer to the wave by a single electron and (2) the degree of microbunching of the electrons. The first factor equals the (negative) work per unit time, *eE*_w_ *v*_T_.

The average value of 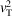, (*Kc*/γ)^2^/2, implies |*v*_T_| ∝ *B*_o_ *P*/γ. Electromagnetism tells us that |*E*_w_| is proportional to the square root of the wave intensity, thus
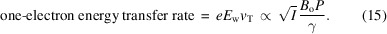
As to the degree of microbunching, we can assume (Margaritondo & Ribic, 2011 ▸; Ribic & Margaritondo, 2012*a* ▸,*b* ▸) that it is approximately given by the longitudinal shift Δ*z* of the electrons inside the bunch divided by the maximum shift, which is λ/2 ≃ *P*/(4γ^2^),
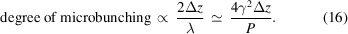
To find Δ*z*, we can use the longitudinal Newton-like equation for the ‘ponderomotive’ force,
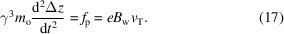
Using again the average value of *v*_T_, ∝ *Kc*/γ ∝ *B*_0_ *P*/γ and the fact that *B*_w_ is also proportional to the square root of the wave intensity, we obtain from equation (17)

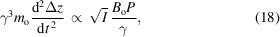
and, using equation (14) as empirical evidence,
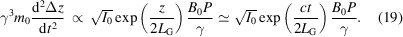
The solution of this differential equation is complicated by the fact that γ is a function of time. However, the experiments show that its changes are small and we can consider it as approximately constant, so equation (19) gives
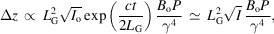


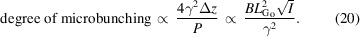
Combining now the two factors of equations (15) and (20), we obtain for the rate of increase of the wave intensity,
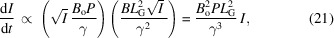
which is a differential equation whose solution is an exponential function of time and therefore of *z* = *vt* ≃ *ct*, self-consistent with equation (14) as long as
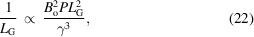


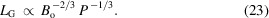
This result justifies, for the most important factors in the gain length, the same roles predicted by much more sophisticated theories (Bonifacio *et al.*, 1994 ▸; Brau, 1990 ▸; Dattoli *et al.*, 1995 ▸).

What causes the end of the intensity growth (Fig. 11 ▸)? After a certain distance *z* the electrons are completely microbunched, and the optical amplification slows down. Furthermore, the loss of energy to the wave decelerates the electrons, decreasing their γ-factor. This changes the emitted wavelength [equations (5) and (6)], so the electrons no longer contribute to the amplified wave.

The actual saturation mechanism is more complicated than this description (Bonifacio *et al.*, 1994 ▸; Brau, 1990 ▸; Dattoli *et al.*, 1995 ▸; Margaritondo & Ribic, 2011 ▸; Ribic & Margaritondo, 2012 ▸) and can produce energy oscillations between electrons and wave, but the result is still the end of the optical amplification. This occurs at a distance (called ‘saturation length’) – approximately 22 times *L*_G_ (Bonifacio *et al.*, 1994 ▸; Brau, 1990 ▸; Dattoli *et al.*, 1995 ▸; Margaritondo & Ribic, 2011 ▸; Ribic & Margaritondo, 2012*a* ▸). Here is why a very long wiggler is needed to fully exploit the optical amplification mechanism.

### 5.3. A historical puzzle?   

Why was the X-FEL technology very difficult to implement, so it was realized only several decades after infrared FELs (Madey, 1971 ▸)? This seems a paradox, since microbunching requires shifting the electrons inside their bunch over a distance comparable to the wavelength. The X-ray wavelengths are much shorter than the infrared ones, thus microbunching would appear easier for X-FELs.

This apparent puzzle is solved in part by considering that the shifts of the electrons by the ‘ponderomotive’ microbunching forces depend on the relevant electron mass, *i.e.* the longitudinal relativistic mass γ^3^ *m*_0_. Since the emission of X-rays requires a large γ [equations (5) and (6)], this mass is very big. In essence, the microbunching mechanism of X-FELs must move extremely ‘heavy’ electrons: even short shifts are difficult.

Furthermore, the short periodicity makes the microbunching very delicate and easily destroyed. And the strong amplification needed for one-pass lasing imposes exceptional characteristics of the electron bunch, including a very small size and a very high density. These and several other technical requirements constitute formidable challenges that explain the long time taken to realize X-FELs.

And they also explain two other facts: first, why normal wigglers and undulators in synchrotron radiation facilities, which do not meet such requirements, do not behave like free-electron lasers. Second, why X-FELs use LINACS rather than storage rings as accelerators.

Indeed, in a storage ring, the transverse cross section of an electron bunch is caused by the slightly different trajectories of its electrons. Such trajectories are influenced by the random emission of synchrotron radiation photons as the electrons circulate around the ring. On the contrary, an electron bunch passes only once through a LINAC with no previous history of synchrotron radiation emission. This allows achieving the small beam cross section and the very high density required for X-FELs.

### 5.4. The exceptional properties of X-FELs   

The main X-FEL characteristic is the high brightness produced by the optical amplification. One must distinguish, however, between average and peak brightness. The emission of an X-FEL consists of short pulses, each corresponding to the passage of an electron bunch through the wiggler. The peak brightness of a pulse is extremely high. But since the pulses are separated by long ‘dead’ times, the average brightness is lower.

**Figure 12:**
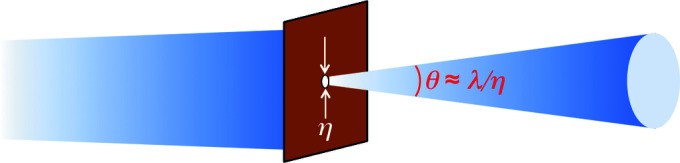
The ‘diffraction limit’: when trying to obtain a small-area source using a shield with a pinhole, diffraction increases the angular spread θ. This limits to ∼1 the increase of the coherent power factor of equation (11).

Note that the brightness increase cannot go beyond the so-called ‘diffraction limit’ (Margaritondo, 1988 ▸; Margaritondo & Ribic, 2011 ▸; Ribic & Margaritondo, 2012*a* ▸,*b* ▸), a property that can be explained in simple terms. Consider the ‘brute force’ way of Fig. 12 ▸ to obtain a source with small size and high lateral coherence: radiation from a large-size source passes through a pinhole in a shield, which becomes a small-area source. This approach is wasteful, since the shield blocks much of the radiation. And it cannot increase the lateral coherence beyond a certain limit: as the pinhole becomes smaller, it diffracts the radiation increasing the angular spread θ.

Thus, the ‘coherent power factor’ λ^2^/(ΩΣ) cannot exceed a natural maximum. To find this maximum, we must consider again the quantum nature of the electromagnetic radiation. The pinhole size η sets the uncertainty in the transverse photon position. And the uncertainty in the photon momentum (of magnitude *h*/λ) is ∼(*h*/λ)θ, so that Heisenberg’s principle gives η(*h*/λ)θ ≃ *h*, and λ/(ηθ) ≃ 1. Since Ω ≃ θ^2^ and the pinhole source size Σ ≃ η^2^, the maximum for the ‘coherent power factor’ λ^2^/(ΩΣ) is ∼1.

This ‘diffraction limit’ is a fundamental property of nature that cannot be overcome by mere technical improvements. And equation (8) shows that it affects the brightness through the geometric parameters Ω and Σ.

Many advanced synchrotron sources are now reaching the ‘diffraction limit’ for such parameters, at least in part of their emitted spectrum. And X-FELs go one step further, by also dramatically increasing the emitted flux – and therefore the brightness – with optical amplification.

Quantitatively, an X-FEL reaching the diffraction limit and effectively exploiting optical amplification can surpass the peak brightness of a synchrotron source by nine orders of magnitude or more (Bonifacio *et al.*, 1994 ▸; Brau, 1990 ▸; Dattoli *et al.*, 1995 ▸; Margaritondo & Ribic, 2011 ▸; Ribic & Margaritondo, 2012*a* ▸). Its average brightness is ‘only’ 100–1000 times larger, but this is already a remarkable increase. Such brightness levels open the door to new classes of experiments, as explained below.

In principle, the peak brightness of X-FELs can be further augmented by improving critical parameters such as the geometry of the electron beam. However, with the extreme density in the microbunches the electron–electron inter­actions, which we neglected, can play a significant role limiting the optical amplification.

Another important property of X-FELs is the time structure. The emission occurs in very short pulses, whose duration corresponds to the extremely small length of each electron bunch, required for one-pass lasing. Typical values range from a fraction of femtosecond to tenths of picoseconds, and allow investigating very important dynamic processes on the same time scales.

These include, for example, fast chemical reactions. Also, in 10^2^ fs, shock waves propagate in solids over a distance comparable to one atom, and ∼10 fs is the time in which a water molecule dissociates.

The most attractive application of super-short, ultra-bright X-FEL pulses is the one-shot structure determination of macromolecules and nanoparticles. At present, many molecular structures are identified by X-ray crystallography – which gathers information simultaneously on many molecules arranged in a periodic lattice. This offsets the problems caused by X-ray-induced damage of individual molecules. But obtaining molecular crystals is often difficult or impossible.

With a short and very bright X-FEL pulse, one could use instead X-ray diffraction to determine the structure of an individual molecule. The extreme pulse energy causes the molecule to explode. But if the pulse is short compared with the explosion time, the information could be extrapolated to the initial structure. This very attractive possibility has been positively tested in selected cases. What will be its ultimate impact? Only the future will tell us, but in principle could be enormous; and with important practical consequences, for example on drug development.

### 5.5. Seeded X-FELs   

Do X-FELs possess coherence? The answer is clearly positive for spatial (lateral) coherence: X-FELs reach the diffraction limit.

The situation is more complex for longitudinal (time) coherence, which requires a narrow wavelength bandwidth. The mechanism described in Sections 5.1 and 5.2 – called ‘self amplified spontaneous emission’ (SASE) (Kondratenko & Saldin, 1980 ▸; Bonifacio *et al.*, 1984 ▸, 1994 ▸) – produces instead a broad bandwidth.

In fact, it starts with the random emission of waves by electrons as they enter the wiggler. After amplification, this produces time-dependent pulses whose lineshape changes from pulse to pulse. According to the Fourier theorem, this corresponds to broad bandwidths of frequencies and wavelengths.

To obtain narrow bandwidths, one can use an external source to produce pulses of well defined shape, and inject them in the wiggler where they are amplified. This is called ‘seeding’ the X-FEL (Feldhaus *et al.*, 1997 ▸; Saldin *et al.*, 2001 ▸; Margaritondo & Ribic, 2011 ▸; Ribic & Margaritondo, 2012*a* ▸). A mere theoretical notion for many years, seeded X-FELs were recently implemented (Togashi *et al.*, 2011 ▸; Allaria *et al.*, 2012 ▸; Amann *et al.*, 2012 ▸; Emma *et al.*, 2017 ▸; Inoue *et al.*, 2019 ▸), yielding high longitudinal coherence. This is particularly important for time-resolved experiments in which the X-FEL pulse provides the ‘start’ time for analyzing fast phenomena.

## 6. Final remarks   

The current evolution of X-FELs opens up new perspectives besides practical applications, touching very interesting fundamental issues. These notably concern the quantum nature of X-rays (Stöhr, 2019 ▸).

As mentioned, in quantum electrodynamics the photon and wave natures of electromagnetic radiation do not coexist: only photons are real. What causes, then, wave-like phenomena like interference and diffraction? Obviously, they must be produced by interactions involving photons.

To clarify this point, consider an experiment in which diffraction or interference is revealed by a fringe pattern. Suppose that the photon flux is low, and on the average only one photon is present at any time in the apparatus. The pattern is still produced as the cumulative result of many photons. This reveals that the interactions causing wave-like phenomena are not between different photons, but of each photon with itself (Stöhr, 2019 ▸) – as Dirac realized very early (Dirac, 1958 ▸).

Such photon self-interactions correspond to the first order of quantum electrodynamics. In visible optics, with sufficient brightness one can also detect higher-order interactions, which can be exploited for very interesting new experimental techniques (Stöhr, 2019 ▸).

Could this be done for X-rays? So far, the answer was negative since the source brightness was not sufficient. But the new seeded X-FELs are changing the situation. Higher-order quantum electrodynamics phenomena are becoming detectable (Stöhr, 2019 ▸) for X-rays, with fundamental as well as practical implications. This is a most exciting new chapter in X-ray science.

## 7. Teaching notes   

Since this article specifically targets teaching, we would like to propose some didactic suggestions based on our own experience.

First, we do not recommend expanding the mathematical formalism, since we found that the level used here can be handled by students from most disciplines.

Second, we also recommend limiting the relativistic notion to those introduced in the first part of the article, for the same reason.

Third, we advise showing example of experimental results, possibly from the teacher’s own research, with emphasis on the most spectacular ones. In that regard, imaging techniques can provide good and attractive choices.

Fourth, we suggest including some historical notes. However, they should be limited to a few of the most relevant milestones in the history of this field. Lastly, the teacher may want to quote references for the descriptions of results that demonstrated the properties treated in this paper or marked the historical breakthroughs. This is a reasonable list:

(i) The original formulation of the synchrotron radiation theory (Iwanenko & Pomeranchuk, 1944 ▸; Schwinger, 1946 ▸; Schwinger, 1949 ▸).

(ii) First experimental detection of synchrotron radiation (Elder *et al.*, 1947 ▸; Pollock, 1983 ▸).

(iii) Early measurements of spectra and other properties of synchrotron radiation (Tomboulian & Hartman, 1956 ▸; Balzarotti *et al.*, 1970 ▸).

(iv) Early experiments using synchrotron radiation (Codling, 1997 ▸; Madden & Codling, 1963 ▸; Sagawa *et al.*, 1966 ▸; Sasaki, 1997 ▸, 2016 ▸; Cauchois *et al.*, 1963 ▸; Balzarotti *et al.*, 1974 ▸; Savoia, 1988 ▸; Perlman *et al.*, 1974 ▸; Kulipanov & Skrinksy, 1988 ▸; Kulipanov *et al.*, 2016 ▸; Hartman, 1988 ▸; Winick & Doniach, 1980 ▸; Bathow *et al.*, 1966 ▸; Haensel *et al.*, 1966 ▸; Steinmann & Skibowski, 1966 ▸).

(v) The transition from parasitic use to dedicated synchrotron radiation sources (Lynch *et al.*, 2015 ▸; Miyahara *et al.*, 1976 ▸).

(vi) The introduction of insertion devices (Winick *et al.*, 1981 ▸; Halbach, 1986 ▸).

(vii) The original proposal of free-electron lasers (Madey, 1971 ▸).

(viii) Theory of X-ray free-electron lasers (Bonifacio *et al.*, 1984 ▸, 1994 ▸: Pellegrini, 2012 ▸).

(ix) The realization of the first hard-X-ray free-electron laser (Emma *et al.*, 2010 ▸).

## Figures and Tables

**Figure 13:**
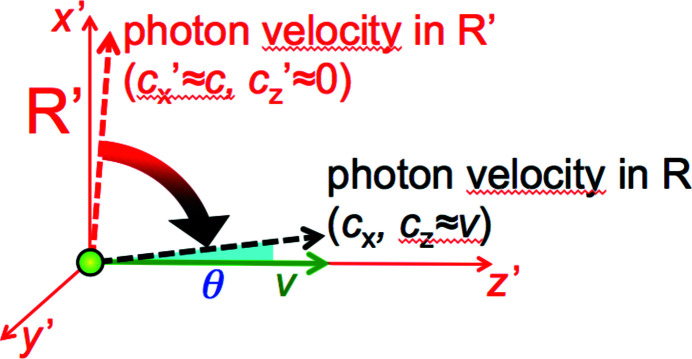
Analysis of the relativistic ‘beaming’.

## Footnotes

1 The Doppler effect was discovered by Christian Doppler half a century before Einstein’s relativity – but it brought him to a tragic end. His work was ferociously attacked by the incompetent colleague Joseph Petzval and his accomplices, with tactics closer to the inquisition than to a scientific debate. This led the University of Vienna to disgracefully dismiss Doppler from its faculty. He died shortly afterwards of tuberculosis while self-exiled in Venice. But his great legacy lives on, as the foundation of all synchrotron radiation experiments of today!

## Appendix A Justifying the Lorentz transformations

In classical physics, the transformation of the *z*-coordinate is simply *z* = *z*′ + *vt*. But there is a problem: divided by *t*, this equation gives *z*/*t* = *z*′/*t* + *v*, so the velocity of an object changes by *v* between the two frames. In the case of light, this would give *c* = *c*′ + *v*, in conflict with the first postulate of relativity. And also in conflict with electromagnetism, which predicts *c* ≃ 3 × 10^8^ m s^−1^ for all frames, a result that was experimentally verified very many times.

The problem disappears with equations (2). Dividing the first equation by the second, we obtain

if *z*′/*t*′ is the speed of light *c* in the R′-frame, this equation gives

consistent with the invariance of *c*.

## Appendix B Doppler shift

The argument of a wavefunction cannot be different in the R-frame and the R′-frame. Otherwise, changes in wave-like phenomena could be used to detect the relative motion of the two frames, violating the second relativistic postulate. Taking into account the direction of propagation and using the speed of light, the arguments of the backscattered wavefunctions in the two frames are

Applying the Lorentz transformations of equations (2) to the first argument, we obtain

equivalent to the second argument as long as

which for *v* ≃ *c* approximately gives equation (5).

## Appendix C How the electrons move in an undulator

In the previous sections, we considered only one interaction: the transverse Lorentz force caused by the longitudinal electron velocity and by the undulator magnetic field. This is the force that causes the oscillating transverse velocity *v*_T_. But the overall picture is more complex, and includes other interactions affecting both *v*_T_ and the longitudinal velocity, which we shall hereafter call *v*_L_.

In fact, the undulator magnetic field, combined with the transverse velocity *v*_T_, also produces a *longitudinal* Lorentz force that changes *v*_L_. This change maintains, as we mentioned above, a constant kinetic energy. In addition, the electrons are subject to the forces of the electric and magnetic fields of the emitted waves. Finally, there are forces between electrons.

We shall neglect this last interaction, which becomes relevant only for electron bunches of very high density. And we shall set aside for now the forces caused by the waves, which will become important later, when discussing free-electron lasers. But we must consider now the longitudinal Lorentz force caused by the undulator and by *v*_T_.

To describe the effects of each force, we shall use the corresponding Newton-like equation linking it to the acceleration. This requires replacing the rest mass *m*_o_ with an appropriate relativistic mass. For the transverse direction, the Newton-like equation is

where *p*_T_ = γ*m*_o_ *v*_T_ is the transverse momentum. In terms of the acceleration d*v*_T_/d*t*, this gives

so the transverse relativistic mass is γ*m*_o_. For the longitudinal momentum *p*_L_ = γ*m*_o_ *v*_L_ one must take into account that the velocity defining γ is the longitudinal one, so

so that

which uses the *longitudinal* relativistic mass γ^3^ *m*_o_.

Let us consider the Newton-like equation for the transverse forces caused by *v*_L_ and *B*. In first approximation, we shall assume that, since , *v*_L_ is almost constant and ∼*c*, so

and

whose solution is

Note that the average of the cosine square is 1/2, therefore the average of  is simply *K* ^2^ *c* ^2^/(2γ^2^).

Let us now consider the longitudinal Newton-like equation linking *v*_L_ and the Lorentz force *f*_L_ created by *B* and *v*_T_,

whose solution (taking into account that the electron has negative charge) is

where the ‘constant’ is the longitudinal velocity outside the undulator, *v*, where *v*_T_ is zero. So, *v*_L_ not only is smaller than *v*, but also slightly oscillates. The root-mean-square average of the magnitude of *v*_L_ is *v* − *K* ^2^ *c*/(4γ^4^).

We can now evaluate the modified factor 1/γ^2^ that changes equation (5) into equation (6),

Using the average of , [*v* − *K* ^2^ *c*/(4γ^4^)]^2^, this factor is, also on average,

Since γ^4^ is large and *K* ^2^ *c*/(4γ^4^) is much smaller than *v* ≃ *c*, we have

and the modified factor 1/γ^2^ is

in agreement with equations (6) and (7).

## Appendix D Doppler ‘beaming’

Suppose (Fig. 13 ▸) that an electron moving at speed *v* ≃ *c* emits a photon in a near-transverse direction close to *x*′ when observed in the electron R′-frame. The longitudinal component of the photon velocity is ∼zero and the transverse component ∼*c*. Look now at the photon velocity in the laboratory R-frame. The source motions ‘projects’ it forward in the longitudinal direction. Since *c* is the same in R and R′, the corresponding change of the vector velocity is a rotation that conserves its magnitude.

The almost transverse emission in R′ thus becomes almost longitudinal in R. By how much? Suppose that the longitudinal component of the photon velocity in R is almost entirely due to the source motion: *c*_*z*_ ≃ *v*. The angle between the photon direction and *z* is θ ≃ *c*_*x*_/*c*_*z*_ ≃ *c*_*x*_/*v*, where *c*_*x*_ is the transverse component of the photon velocity. But  =  +  ≃  + , so

Thus, the angular range of the emission, 2θ, is indeed of the order of 2/γ.

## Appendix E Ponderomotive forces

We have seen in Appendix *C* [App appc] that

giving

We can now consider the force of magnitude *eE*_w_ of the wave electric field, which slightly changes *v*_T_ according to the transverse Newton-like law,

The change d*v*_T_ also modifies *v*_L_,

corresponding to a contribution to the time derivative d*v*_L_/d*t* of magnitude

since for a wave *E*_w_/*c* = *B*_w_, this result proves the presence of a longitudinal (‘ponderomotive’) force of magnitude given by equation (12), acting on an electron with longitudinal relativistic mass γ^3^ *m*_0_.
